# Mental health, childhood abuse and HIV sexual risk behaviour among university students in Ivory Coast

**DOI:** 10.1186/1744-859X-12-18

**Published:** 2013-06-11

**Authors:** Karl Peltzer, Supa Pengpid, Issaka Tiembre

**Affiliations:** 1 HIV/AIDS, STI and TB (HAST) Research Programme, Human Sciences Research Council, Private Bag X41, Pretoria, South Africa; 2 Department of Psychology, University of Limpopo, Turfloop, South Africa; 3 ASEAN Institute for Health Development, Madidol University, Salaya, Phutthamonthon, Nakhonpathom 73170, Thailand; 4 Institut National d'Hygiène Publique, Université Félix Houphouët Boigny de Cocody, Abidjan, Bp V 14, Ivory Coast

## Abstract

**Background:**

Little focus has been paid to the role of poor mental health and childhood abuse among young people with regard to human immunodeficiency virus (HIV) risk behaviour and HIV prevention in Africa. The aim of this study was to determine the association between mental health, childhood abuse and HIV sexual risk behaviour among a sample of university students in Ivory Coast.

**Methods:**

A cross-sectional survey was conducted with undergraduate students that were recruited randomly from classes at the Félix Houphouët Boigny University of Cocody. The sample included 824 university students (50% men and 50% women), with a mean age of 23.7 years (SD = 2.7).

**Results:**

Of the 824 university students who completed the survey, 17.6% reported depression, 10.8% screened positive for post-traumatic stress disorder, 8.3% reported at least monthly heavy episodic drinking, 13.5% reported childhood physical abuse and 4.7% sexual abuse, 33.9% had two or more sexual partners in the past 12 months, 66.3% had inconsistent condom use, 23.6% had alcohol use in the context of sex and 16.7% had a history of a sexually transmitted infection In multivariable analysis among men, lack of religiousness and alcohol use in the context of sex were associated with HIV risk behaviour, and among women, poorer family background, experience of sexual and physical partner violence, alcohol use in the context of sex and depression were associated with HIV risk behaviour.

**Conclusions:**

Poor mental health (depression) including alcohol use and partner violence was found to be associated with HIV risk behaviour. Coordinated mental health and sexual and reproductive health services to meet the needs of university students would be desirable.

## Introduction

The infection rate of human immunodeficiency virus (HIV)/acquired immunodeficiency syndrome (AIDS) in Ivory Coast is estimated at 7.1% in adults ages 15–49. Ivory Coast has a generalised HIV epidemic with the highest prevalence rate in the West African region. Populations at comparatively high risk for HIV infection include women ages 20–24 and youth [1]. High HIV risk behaviour has been reported among youth in Abidjan, Ivory Coast [2].

Poor mental health is a major cause of morbidity in middle-income countries, with depression constituting the heaviest disease burden [3]. The HIV epidemic contributes to increased depression rates both in persons who live with HIV [4] and in those who are indirectly affected by HIV/AIDS [5]. Several reviews in the general population indicate that there may be a relationship between poor mental health including substance use and childhood abuse and HIV risk behaviour such as multiple sexual partners and unprotected sex [6-15].

Studies with university students found that anxiety [16], depression [16,17], psychological distress [17], alcohol use [17-22], drug use [21,22] and traumatic experiences [23], in particular childhood abuse, physical abuse and especially sexual abuse [24], were associated with various forms of HIV risk behaviour. Currently, there is no sufficient data on university students regarding the association between mental disorders, childhood abuse and HIV sexual risk behaviour within the West African context. Therefore, the aim of this study was to determine the association between mental health, childhood abuse and HIV sexual risk behaviour among Félix Houphouët Boigny University undergraduate students in Ivory Coast.

## Methods

### Sample and procedure

A cross-sectional survey design was used. An anonymous, self-administered questionnaire was used to collect data. Concerning the sampling procedure, we conducted a simple random sampling, stratified and weighted on the size of units of training and research (UTR) of Félix Houphouët Boigny University. Indeed, from the size of the different units of training and research (faculties), we determined the percentage weight of each unit training and research (faculty) compared to the total number of students at the Félix Houphouët Boigny University. Then, we assigned this percentage to 800 students in question, which gave us the number of student per unit training and research or faculty. In each UTR, we conducted a simple random sample rate. Permission to carry out the study was obtained from the President of Félix Houphouët Boigny University and ethical clearance was also taken. The cover pages of the questionnaires briefly explained about the study and provided instructions to the respondents on how to fill it up. It also provided information about the researchers. It also mentioned that anonymity and confidentiality would be maintained and that the participation of students was voluntary. It specified that data would be used only for research purposes. Written informed consent was taken from participating students, and the study was conducted from 7 January to 8 February 2013.

### Measures

#### Traumatic experiences

Participants were asked if they had ever been hit by a sex partner, forced to have sex, physically abused as a child, sexually abused as a child and diagnosed as HIV positive. Traumatic experience items were coded as yes/no [25].

#### Post-traumatic stress disorder

A seven-item screener (*α* = 0.77) was used to identify post-traumatic stress disorder (PTSD) symptoms in the past month [25,26]. Items asked whether the respondent had experienced difficulties related to a traumatic experience (e.g., ‘Did you begin to feel more isolated and distant from other people?’ and ‘Did you become jumpy or get easily startled by ordinary noises or movements?’). Consistent with epidemiological evidence, participants who answered affirmatively to at least four of the questions were considered to have a positive screen for PTSD [25,26].

#### Depressive symptoms

We assessed *depressive symptoms* using the ten-item version of the Center for Epidemiologic Studies Depression (CES-D) scale [27]. While the CES-D ten-item survey has not been directly compared to clinical diagnosis of major depression, the sensitivity and specificity of the CES-D 20-item survey has been reported to average 80% and 70%, respectively, compared to formal diagnostic interview [28]. Scoring is classified from 0 to 9 as having mild level of depressive symptoms, 10 to 14 as moderate depressive symptoms, and ≥15 representing severe depressive symptoms [29]. The Cronbach *α* reliability coefficient of this ten-item scale was 0.72 in this study.

#### Alcohol consumption

*Alcohol* consumption was measured by asking participants which of the following terms best described them: non-drinker, special occasion drinker, occasional and regular drinker. Occasional and regular drinkers were asked, ‘how often do you have (for men) five or more and (for women) four or more drinks on one occasion?’ Response options ranged from 1 = never to 5 = daily or almost daily.

HIV risk behaviour items were developed based on literature review [25,30,31] and assessed four components of HIV risk, number of sexual partners, protected or unprotected sexual intercourse, alcohol use in the context of sex and history of a sexually transmitted infection (STI). HIV risk behaviour, therefore, was assessed with four items, number of sexual partners in the past 12 months, condom use consistency in the past 3 months with primary partner, alcohol use in the context of sex in the past 3 months and ever having been diagnosed with an STI.

Socioeconomic background was assessed by having students rate their family background as wealthy (within the highest 25% in Ivory Coast, in terms of wealth), quite well off (within the 50% to 75% range for their country), not very well off (within the 25% to 50% range from Ivory Coast) or quite poor (within the lowest 25% in their country, in terms of wealth). We subsequently divided the students into poorer (not very well off and quite poor) and wealthier (wealthy, quite well off) categories.

#### Religiousness

*Religiousness* was assessed with the five-item Duke University Religion Index (DUREL) [32]. The instrument assesses the three major dimensions of religiosity: organizational religious activity, non-organizational religious activity, and intrinsic religiosity (or subjective religiosity) [33]. The DUREL measures each of these dimensions by a ‘separate subscale’ , and correlations with health outcomes are analysed by subscale in separate models [33]. Cronbach alpha for the intrinsic religiosity scale was 0.60 for this sample.

### Data analysis

The data were analysed using IBM SPSS (version 20.0). First, gender differences were analysed for all variables using chi-square tests. Since there were significant gender differences on a number of variables, subsequent models were analysed separately for men and women. Second, logistic regression analyses were done to identify traumatic experiences that were in bivariate analysis associated with a positive screen for PTSD and a positive screen for depression [25]. Third, logistic regression was used to identify factors that were associated with HIV risk behaviour. Predictor variables were entered in a single step.

## Results

### Sample characteristics

Of the 860 students approached for the study, 824 agreed to participate in the survey (96% response rate). The sample included 824 university students (50% men and 50% women), with a mean age of 23.7 years (SD = 2.7), 43.2% studying in their first year, 22.8% second year, 21.6% third year and 12.4% fourth year, and 82.9% were from a poorer family background. Most students were, by religious affiliation, Christians (77.6%) and Muslims (17.5%). Regarding mental health, 17.6% reported depression, 10.8% screened positive for PTSD and 8.3% reported at least monthly heavy episodic drinking. Childhood physical abuse was 13.5% and sexual abuse 4.7%. High HIV risk behaviour was reported: 33.9% had two or more sexual partners in the past 12 months, 66.3% had inconsistent condom use, 23.6% had alcohol use in the context of sex and 16.7% had a history of a STI. Both women and men indicated that they were subjected to partner violence, with 21.2% of women reporting sexual violence. Few (1.1%) indicated that they were HIV positive (see Table 1).

**Table 1:** Sample characteristics

**Variable**	**Total (*n*= 824)**		**Men (*n*= 412)**		**Women (*n*= 412)**		**Statistic**
	**Number**	**SD (%)**	**Number**	**SD (%)**	**Number**	**SD (%)**	
Socio-demographics							
Age (years)							
18–22	272	33.0	114	27.7	158	38.3	0.005
23–25	325	39.4	177	43.0	148	35.9	
26–30	227	27.5	121	29.4	106	25.7	
Family background							
Quite poor	162	20.6	108	27.7	54	13.7	<0.001
Not very well off	489	62.3	236	60.5	253	64.1	
Quite well off, wealthy	134	17.1	46	11.8	88	22.3	
Religious affiliation							
Muslim	144	17.5	72	17.5	72	17.5	<0.001
Protestant	301	36.5	169	41.0	132	32.0	
Catholic	339	41.1	140	34.0	199	48.3	
Others	40	4.9	31	7.5	9	2.2	
Religiousness							
Organised religious activity	4.9	1.3	4.8	1.4	5.0	1.2	0.027
Non-organised religious activity	3.5	1.9	3.3	1.9	3.8	1.8	<0.001
Intrinsic religiosity	16.2	2.1	15.9	2.2	16.4	1.9	<0.001
Traumatic experiences							
Ever been hit by a sex partner	53	7.2	14	3.9	39	10.3	0.001
Ever been forced to have sex	109	14.7	29	7.9	80	21.2	<0.001
Physically abused as a child	99	13.5	65	17.7	34	9.2	0.001
Sexually abused as a child	35	4.7	14	3.8	21	5.6	0.258
HIV positive	8	1.1	4	1.1	4	1.1	0.997
Mental health							
Screened positive for depression	143	17.6	63	15.4	80	19.8	0.228
Screened positive for PTSD	88	10.8	38	9.4	50	12.3	0.240
Heavy episodic drinking	68	8.3	55	13.3	13	3.2	<0.001
HIV risk							
Two or more sexual partners	261	33.9	177	45.5	84	22.1	<0.001
Inconsistent condom use	430	66.3	207	61.8	223	71.0	0.013
Alcohol in the context of sex	171	23.6	110	30.0	61	17.1	<0.001
History of STI	122	16.7	50	13.8	72	19.6	0.036

#### Predictors of multiple sexual partners

In bivariate analysis among men, lack of organised and non-organised religious activities, lack of intrinsic religiosity, heavy episodic drinking, alcohol use in the context of sex and having screening positive for depression were associated with two or more sexual partners in the past 12 months. In multivariate analysis among men, lack of non-organised religious activity and alcohol use in the context of sex were associated with two or more sexual partners in the past 12 months. Further, in bivariate analysis among women, poorer family background, lack of non-organised religious activity, lack of intrinsic religiosity, experience of physical and sexual partner violence and alcohol use in the context of sex were associated with two or more sexual partners in the past 12 months. In multivariate analysis among women, poorer family background, experience of sexual partner violence and alcohol use in the context of sex were associated with two or more sexual partners in the past 12 months. Among both men and women, childhood physical and sexual abuse was not found to be associated with multiple sexual partners (see Table 2).

**Table 2:** Logistic regression analyses predicting multiple sexual partners

**Variable**	**Men**		**Women**	
	**Crude odds ratio (95% CI)**	**Adjusted odds ratio (95% CI)**	**Crude odds ratio (95% CI)**	**Adjusted odds ratio (95% CI)**
Socio-demographics				
Age (years)				
18–22	1.00	–	1.00	–
23–25	1.30 (0.80–2.13)	–	1.72 (0.96–3.06)	–
26–30	1.16 (0.69–1.97)	–	1.63 (0.86–3.08)	–
Family background				
Quite poor	1.00	–	1.00	1.00
Not very well off	0.99 (0.61–1.59)	–	0.52 (0.27–1.00)*	0.45 (0.20–1.02)
Quite well off, wealthy	0.86 (0.42–1.76)	–	0.47 (0.21–1.05)	0.35 (0.12–0.99)*
Religious affiliation				
Muslim	1.00	–	1.00	–
Protestant	0.59 (0.34–1.05)	–	0.59 (0.27–1.29)	–
Catholic	1.26 (0.71–2.25)	–	1.39 (0.71–2.72)	–
Others	0.93 (0.39–2.18)	–	1.21 (0.22–6.69)	–
Religiousness				
Organised religious activity	0.78 (0.67–0.96)***	1.11 (0.84–1.45)	0.86 (0.70–1.05)	–
Non-organised religious activity	0.79 (0.70–0.89)***	0.79 (0.65–0.97)*	0.87 (0.76–1.00)*	0.88 (0.75–1.05)
Intrinsic religiosity	0.54 (0.35–0.83)**	0.59 (0.31–1.13)	0.56 (0.33–0.95)*	0.73 (0.39–1.39)
Traumatic experiences				
Ever been hit by a sex partner	1.81 (0.58–5.66)	–	2.04 (1.00–4.21)*	1.21 (0.48–3.06)
Ever been forced to have sex	1.34 (0.62–2.91)	–	2.99 (1.72–5.19)***	2.53 (1.25–5.12)**
Physically abused as a child	0.96 (0.56–1.66)	–	1.12 (0.48–2.59)	–
Sexually abused as a child	1.54 (0.53–4.56)	–	1.52 (0.56–4.10)	–
Diagnosed HIV positive	0.00	–	1.17 (0.12–11.36)	–
Alcohol use				
Heavy episodic drinking	2.66 (1.45–4.88)**	2.17 (0.82–5.73)	2.42 (0.67–8.77)	–
Alcohol use in the context of sex	2.62 (1.64–4.18)***	2.78 (1.39–5.58)**	4.13 (2.27–7.50)***	3.07 (1.50–6.29)**
Mental health				
Screened positive for depression	1.88 (1.00–3.48)*	2.24 (0.89–5.65)	1.55 (0.81–2.93)	–
Screened positive for PTSD	0.54 (0.24–1.20)	–	1.36 (0.62–2.98)	–

*P < .05; **P < .01; ***P < .001.

#### Predictors of inconsistent condom use

In bivariate analysis among men, only other religious affiliation was associated with inconsistent condom use in the past 3 months. Further, in bivariate analysis among women, experience of physical and sexual partner violence was associated with inconsistent condom use in the past 3 months. In multivariate analysis among women, experience of physical partner violence remained associated with inconsistent condom use in the past 3 months (see Table 3).

**Table 3:** Logistic regression analyses predicting inconsistent condom use

**Variable**	**Men**	**Women**	
	**Crude odds ratio (95% CI)**	**Crude odds ratio (95% CI)**	**Adjusted odds ratio (95% CI)**
Socio-demographics			
Age (years)			
18–22	1.00	1.00	–
23–25	1.01 (0.59–1.75)	1.16 (0.66–2.04)	–
26–30	1.02 (0.57–1.81)	1.42 (0.76–2.66)	–
Family background			
Quite poor	1.00	1.00	–
Not very well off	1.11 (0.65–1.88)	1.50 (0.74–3.05)	–
Quite well off, wealthy	1.66 (0.74–3.72)	1.42 (0.63–3.20)	–
Religious affiliation			
Muslim	1.00	1.00	–
Protestant	1.86 (0.98–3.51)	2.09 (0.97–4.52)	–
Catholic	1.69 (0.89–3.22)	0.98 (0.51–1.88)	–
Others	3.28 (1.14–9.47)*	0.97 (0.16–5.82)	–
Religiousness			
Organised religious activity	0.93 (0.78–1.10)	1.19 (0.97–1.45)	–
Non-organised religious activity	0.93 (0.82–1.05)	1.09 (0.95–1.26)	–
Intrinsic religiosity	0.83 (0.52–1.32)	1.22 (0.72–2.08)	–
Traumatic experiences			
Ever been hit by a sex partner	1.28 (0.38–4.34)	2.64 (1.00–6.05)*	5.62 (1.29–24.57)*
Ever been forced to have sex	1.53 (0.61–3.80)	1.94 (1.02–3.75)*	1.49 (0.73–3.04)
Physically abused as a child	0.96 (0.54–1.72)	1.03 (0.41–2.57)	–
Sexually abused as a child	3.56 (0.78–16.37)	0.81 (0.29–2.23)	–
Diagnosed HIV positive	0.00	0.00	–
Alcohol use			
Heavy episodic drinking	1.28 (0.68–2.41)	0.56 (0.17–1.80)	–
Alcohol use in the context of sex	1.02 (0.63–1.66)	0.84 (0.45–1.55)	–
Mental health			
Screened positive for depression	0.69 (0.35–1.36)	1.04 (0.54–2.01)	–
Screened positive for PTSD	0.89 (0.39–2.04)	1.13 (0.48–2.68)	–

*P < .05.

#### Predictors of history of STI

In bivariate analysis among men, lack of non-organised religious activity and alcohol use in the context of sex were associated with a history of STI, while in multivariate analysis, lack of non-organised religious activity remained associated with a history of STI. Further, in bivariate analysis among women, alcohol use in the context of sex and having screened positive with depression were associated with a history of STI, while multivariate analysis having screened positive with depression remained associated with a history of STI (see Table 4).

**Table 4:** Logistic regression analyses predicting history of STI

**Variable**	**Men**		**Women**	
	**Crude odds ratio (95% CI)**	**Adjusted odds ratio (95% CI)**	**Crude odds ratio (95% CI)**	**Adjusted odds ratio (95% CI)**
Socio-demographics				
Age				
18–22	1.00	–	1.00	–
23–25	1.54 (0.68–3.51)	–	1.51 (0.82–2.77)	–
26–30	1.85 (0.79–4.30)	–	1.26 (0.65–2.44)	–
Family background				
Quite poor	1.00	–	1.00	–
Not very well off	0.72 (0.37–1.42)	–	0.88 (0.42–1.88)	–
Quite well off, wealthy	0.83 (0.30–2.31)	–	0.49 (0.19–1.25)	–
Religious affiliation				
Muslim	1.00	–	1.00	–
Protestant	1.65 (0.64–4.31)	–	1.08 (0.47–2.47)	–
Catholic	1.50 (0.56–3.99)	–	1.45 (0.68–3.12)	–
Others	1.41 (0.37–5.43)	–	0.00	–
Religiousness				
Organised religious activity	1.00 (0.80–1.24)	–	0.82 (0.66–1.01)	–
Non-organised religious activity	0.84 (0.70–1.00)*	0.82 (0.68–0.99)*	0.90 (0.78–1.04)	–
Intrinsic religiosity	0.77 (0.40–1.47)	–	0.95 (0.55–1.66)	–
Traumatic experiences				
Ever been hit by a sex partner	2.03 (0.53–7.78)	–	0.97 (0.41–2.31)	–
Ever been forced to have sex	1.12 (0.37–3.41)	–	1.75 (0.98–3.13)	–
Physically abused as a child	0.88 (0.39–1.99)	–	1.63 (0.72–3.68)	–
Sexually abused as a child	1.84 (0.49–6.94)	–	1.12 (0.36–3.49)	–
Diagnosed HIV positive	3.05 (0.77–34.34)	–	1.42 (0.15–13.84)	–
Alcohol use				
Heavy episodic drinking	1.53 (0.71–3.28)	–	0.74 (0.16–3.42)	–
Alcohol use in the context of sex	1.96 (1.05–3.66)*	1.67 (0.87–3.19)	1.92 (1.01–3.64)*	1.68 (0.57–3.24)
Mental health				
Screened positive for depression	1.55 (0.67–3.58)	–	2.17 (1.17–4.03)*	1.99 (1.03–3.85)*
Screened positive for PTSD	1.54 (0.59–4.02)	–	1.75 (0.81–3.76)	–

*P < .05.

## Discussion

This study found a high prevalence of poor mental health (depression and PTSD symptoms), high reported childhood physical and sexual abuse and high HIV risk behaviour (multiple sexual partners, unprotected sex, alcohol in the context of sex and history of STI) among a sample of university students in Ivory Coast. The high prevalence of HIV risk behaviour is calling for HIV prevention intervention programs among university students.

The prevalence of depression and PTSD was similar to other studies in Africa [16,34-36]. The study found that poor mental health (depression) including alcohol use was associated with HIV risk behaviour among this sample of university students in Ivory Coast. This finding is in concordance with previous studies among university students [16,17,19-23]. While associations between alcohol use [17-22] and HIV risk behaviours have previously been found among university students, the mechanisms explaining the association between alcohol use and HIV risk behaviours in this setting are not fully understood [17]. Alcohol use in the context of sex and binge drinking seem to be associated in particular with indiscriminate forms of risky sex (e.g., having multiple or casual sex partners), which is keeping with the studies of American college populations [37,38].

The prevalence of childhood physical and sexual abuse was in this study (17.7% and 9.2% physical abuse among men and women, respectively, and 3.8% and 5.6% sexual abuse among men and women, respectively) similar to what was found in community samples in South Africa, Tanzania and Zimbabwe (14.9%–27.4% and 4.3%–15.6% physical abuse among men and women, respectively, and 1.6%–4.5% and 2.4%–4.9% sexual abuse among men and women, respectively) [15]. Contrary to some other studies [15,24,39], this study did not find an association between childhood physical and sexual abuse and HIV risk behaviour. The study found that among women, the experience of physical and/or sexual partner violence was associated with HIV risk behaviour. As found in other studies [23,40-44] women who experience intimate partner violence are at risk for HIV through high-risk heterosexual contact. Overall, poor mental health, traumatic experiences and substance use may synergistically interact to increase HIV risk behaviour [26].

The study found that among men, lack of religiousness was associated with HIV risk behaviour (multiple sexual partners and history of STI), but it was not associated with condom use. Similarly, in a study among university students in Uganda for those who rated religion as less important in their family, the probability of having a high number of lifetime partners increased significantly, while the role of religion seemed to have no impact on condom use [45]. Religious beliefs and attitudes on sexual matters appear to have considerable influence on the sexual conduct of young men in the Ivory Coast [46] which can be utilised for HIV prevention programs. Finally, women from poorer family backgrounds were more likely to be engaging in sex with multiple partners. It is possible that students from poorer backgrounds engage more likely in transactional sex [47].

### Study limitations

Limitation pertains to the generalizability of the study results, where caution should be taken when interpreting these results, as only full-time undergraduate students between the ages of 18 to 30 years were included in this study. It is unknown to what extents these findings can be generalised to part-time or non-resident students. The data used in the study were obtained by self-report which could have been the result of desired participants' responses. Although the study was anonymous, the sensitive nature of the items related to sexual behaviour could have an impact on the participants' responses. Moreover, this study was based on the data collected in a cross-sectional survey. We cannot, therefore, ascribe causality to any of the associated factors in the study. A further limitation was that certain mental health measures were brief screenings and may be interpreted only as indicators of poor mental health [26,27]. In addition, the study did not screen for the history of severe mental illness, which has been found to be associated with HIV risk behaviour [44], and should be included in future studies.

## Conclusions

High rates of depression, PTSD, childhood abuse and HIV risk behaviour were found among the studied university student population. Poor mental health (depression) including alcohol use and partner violence was associated with HIV risk behaviour. Coordinated mental health and sexual and reproductive health services to meet the needs of university students would be desirable.

## Competing interests

The authors declare that they have no competing interests.

## Authors' contributions

KP was the main contributor to the conceptualization of the study. KP and SP contributed significantly to the first draft of the paper, and KP, SP and IT contributed to the subsequent drafts and finalization. All authors read and approved the final manuscript.

## References

[B1] U.S. Department of State 2008 Country Profile: Cote d'Ivoire 2008 Washington, D.C: U.S. Department of State

[B2] Toure B Koffi K Kouassi-Gohou V Kokoun E Angbo-Effi O Koffi NM Diarra-Nam AJ Awareness, attitudes, and practices of secondary school students in relation to HIV/AIDS in Abidjan Ivory Coast. Med Trop 2005 65 4 346 348 16548487

[B3] Patel V Mental health in low- and middle-income countries Br Med Bull 2007 81–82 81 96 17470476 10.1093/bmb/ldm010

[B4] Ciesla JA Roberts JE Meta-analysis of the relationship between HIV infection and risk for depressive disorders Am J Psychiatry 2001 158 5 725 730 10.1176/appi.ajp.158.5.725 11329393

[B5] Myer L Seedat S Stein DJ Moomal H Williams DR The mental health impact of AIDS-related mortality in South Africa: a national study J Epidemiol Community Health 2009 63 4 293 298 10.1136/jech.2008.080861 19074926 PMC3203694

[B6] Crepaz N Marks G Are negative affective states associated with HIV sexual risk behaviors? A meta-analytic review Health Psychol 2001 20 4 291 299 11515741 10.1037//0278-6133.20.4.291

[B7] Rehm J Shield KD Joharchi N Shuper PA Alcohol consumption and the intention to engage in unprotected sex: systematic review and meta-analysis of experimental studies Addiction 2012 107 1 51 59 10.1111/j.1360-0443.2011.03621.x 22151318

[B8] Rabkin JG HIV and depression: 2008 review and update Curr HIV/AIDS Rep 2008 5 4 163 171 10.1007/s11904-008-0025-1 18838056

[B9] Lloyd S Operario D HIV risk among men who have sex with men who have experienced childhood sexual abuse: systematic review and meta-analysis AIDS Educ Prev 2012 24 3 228 341 10.1521/aeap.2012.24.3.228 22676462

[B10] Chersich MF Rees HV Causal links between binge drinking patterns, unsafe sex and HIV in South Africa: its time to intervene Int J STD AIDS 2010 21 1 2 7 10.1258/ijsa.2000.009432 20029060

[B11] Pithey A Parry C Descriptive systematic review of sub-Saharan African studies on the association between alcohol use and HIV infection SAHARA J 2009 6 4 155 169 10.1080/17290376.2009.9724944 20485855

[B12] Kalichman SC Simbayi LC Kaufman M Cain D Jooste S Alcohol use and sexual risks for HIV/AIDS in sub-Saharan Africa: systematic review of empirical findings Prev Sci 2007 8 2 141 151 10.1007/s11121-006-0061-2 17265194

[B13] Wyatt GE Myers HF Loeb TB Women, trauma, and HIV: an overview AIDS Behav 2004 8 4 401 403 10.1007/s10461-004-7324-3 15690113

[B14] Wilson HW Widom CS Pathways from childhood abuse and neglect to HIV-risk sexual behavior in middle adulthood J Consult Clin Psychol 2011 79 2 236 246 21355638 10.1037/a0022915 PMC3066267

[B15] Richter L Komárek A Desmond C Celentano D Morin S Sweat M Chariyalertsak S Chingono A Gray G Mbwambo J Coates T Reported physical and sexual abuse in childhood and adult HIV risk behaviour in three African countries: findings from Project Accept (HPTN-043) AIDS Behav 2013 10.1007/s10461-013-0439-7 PMC3796176 23474641

[B16] Agardh A Cantor-Graae E Ostergren PO Youth, sexual risk-taking behavior, and mental health: a study of university students in Uganda Int J Behav Med 2012 19 2 208 216 10.1007/s12529-011-9159-4 21590465 PMC3358553

[B17] Lundberg P Rukundo G Ashaba S Thorson A Allebeck P Ostergren PO Cantor-Graae E Poor mental health and sexual risk behaviours in Uganda: a cross-sectional population-based study BMC Publ Health 2011 11 125 10.1186/1471-2458-11-125 PMC3056745 21338500

[B18] Kim J Celentano DD Crum RM Alcohol consumption and sexually transmitted disease risk behavior: partner mix among male Korean university students Alcohol Clin Exp Res 1998 22 1 126 131 9514295

[B19] Griffin JA Umstattd MR Usdan SL Alcohol use and high-risk sexual behavior among collegiate women: a review of research on alcohol myopia theory J Am Coll Health 2010 58 6 523 532 10.1080/07448481003621718 20452928

[B20] Umoh OO Umoh UJ Alcohol related problems, family type and youth HIV/AIDS risk behaviour Afr J Drug Alcohol Studies 2011 10 1 49 58

[B21] Hoque ME Reported risky sexual practices amongst female undergraduate students in KwaZulu-Natal, South Africa Afr J Prm Health Care Fam Med 2011 3 1 6 10.4102/phcfm.v3i1.281

[B22] Adefuye AS Abiona TC Balogun JA Lukobo-Durrell M HIV sexual risk behaviors and perception of risk among college students: implications for planning interventions BMC Publ Health 2009 9 281 10.1186/1471-2458-9-281 PMC2733304 19653901

[B23] Agardh A Odberg-Pettersson K Ostergren PO Experience of sexual coercion and risky sexual behavior among Ugandan university students BMC Publ Health 2011 11 527 10.1186/1471-2458-11-527 PMC3148576 21726433

[B24] Collings SJ Childhood sexual abuse in a sample of South African university males: prevalence and risk factors S Afr J Psychol 1991 21 3 153 158

[B25] Sikkema KJ Watt MH Meade CS Ranby KW Kalichman SC Skinner D Pieterse D Mental health and HIV sexual risk behavior among patrons of alcohol serving venues in Cape Town, South Africa J Acquir Immune Defic Syndr 2011 57 3 230 237 10.1097/QAI.0b013e3182167e7a 21372724 PMC3135683

[B26] Kimerling R Ouimette P Prins A Nisco P Lawler C Cronkite R Moos RH Brief report: utility of a short screening scale for DSM-IV PTSD in primary care J Gen Intern Med 2006 21 1 65 67 10.1111/j.1525-1497.2005.00292.x 16423126 PMC1484617

[B27] Andresen EM Malmgren JA Carter WB Patrick DL Screening for depression in well older adults: evaluation of a short form of the CES-D (Center for Epidemiologic Studies Depression Scale) Am J Prev Med 1994 10 2 77 84 8037935

[B28] Mulrow CD Williams JW Jr Gerety MB Ramirez G Montiel OM Kerber C Case-finding instruments for depression in primary care settings Ann Intern Med 1995 122 12 913 921 10.7326/0003-4819-122-12-199506150-00004 7755226

[B29] Kilbourne A Justice A Rollman B McGinnis K Rabeneck L Weissman S Clinical importance of HIV and depressive symptoms among veterans with HIV infection J Gen Intern Med 2002 17 7 512 520 10.1046/j.1525-1497.2002.10803.x 12133141 PMC1495073

[B30] Kalichman SC Simbayi L Jooste S Vermaak R Cain D Sensation seeking and alcohol use predict HIV transmission risks: prospective study of sexually transmitted infection clinic patients Cape Town. South Africa. Addict Behav 2008 33 12 1630 1633 10.1016/j.addbeh.2008.07.020 PMC4271623 18790575

[B31] Peltzer K Simbayi L Kalichman S Jooste S Cloete A Mbelle N Drug use and HIV risk behaviour in three urban South African communities J Soc Sci 2009 18 2 143 149

[B32] Koenig HG Meador K Parkerson G Religion index for psychiatric research: a 5-item measure for use in health outcome studies Am J Psychiatry 1997 154 885 886 9167530 10.1176/ajp.154.6.885b

[B33] Koenig HG Büssing A The Duke University Religion Index (DUREL): a five-item measure for use in epidemological studies Religions 2010 1 78 85 10.3390/rel1010078

[B34] Aniebue PN Onyema GO Prevalence of depressive symptoms among Nigerian medical undergraduates Trop Doct 2008 38 3 157 158 10.1258/td.2007.070202 18628542

[B35] Adewuya AO Ola BA Aloba OO Mapayi BM Oginni OO Depression amongst Nigerian university students Prevalence and sociodemographic correlates. Soc Psychiatry Psychiatr Epidemiol 2006 41 8 674 678 10.1007/s00127-006-0068-9 16680408

[B36] Peltzer K Traumatic experiencing and post traumatic psychological symptoms in South African University students Cent Afr J Med 1998 44 11 280 283 10189748

[B37] Cooper ML Alcohol use and risky sexual behavior among college students and youth: evaluating the evidence J Stud Alcohol Suppl 2002 14 101 117 12022716 10.15288/jsas.2002.s14.101

[B38] Howard DE Wang MQ The relationship between substance use and STD/HIV-related sexual risk behaviors among U.S. adolescents. J HIV/AIDS Prev Children Youth 2004 6 2 65 82 10.1300/J499v06n02_05

[B39] Homma Y Wang N Saewyc E Kishor N The relationship between sexual abuse and risky sexual behavior among adolescent boys: a meta-analysis J Adolesc Health 2012 51 1 18 24 10.1016/j.jadohealth.2011.12.032 22727072 PMC4829388

[B40] Campbell JC Baty ML Ghandour RM Stockman JK Francisco L Wagman J The intersection of intimate partner violence against women and HIV/AIDS: a review Intern J Inj Control Safety Prom 2008 15 4 221 231 10.1080/17457300802423224 PMC3274697 19051085

[B41] Coker AL Does physical intimate partner violence affect sexual health? A systematic review Trauma Violence Abuse 2007 8 2 149 177 10.1177/1524838007301162 17545572

[B42] Jewkes RK Dunkle K Nduna M Shai N Intimate partner violence, relationship power inequity, and incidence of HIV infection in young women in South Africa: a cohort study Lancet 2010 376 9734 41 8 10.1016/S0140-6736(10)60548-X 20557928

[B43] Wu E El-Bassel N Witte SS Gilbert L Chang M Intimate partner violence and HIV risk among urban minority women in primary health care settings AIDS Behav 2003 7 3 291 301 10.1023/A:1025447820399 14586191

[B44] Meade CS Sikkema KJ HIV risk behavior among adults with severe mental illness: a systematic review Clin Psychol Rev 2005 25 4 433 57 10.1016/j.cpr.2005.02.001 15914265

[B45] Agardh A Tumwine G Östergren PO The impact of socio-demographic and religious factors upon sexual behavior among Ugandan university students PLoS One 2011 6 8 e23670 10.1371/journal.pone.0023670 21887292 PMC3161050

[B46] Koffi AK Kawahara K Sexual abstinence behavior among never married youths in a generalized HIV epidemic country: evidence from the 2005 Cote d’Ivoire AIDS indicator survey BMC Publ Health 2008 8 408 10.1186/1471-2458-8-408 PMC2628901 19087306

[B47] Masvawure T ‘I just need to be flashy on campus’: female students and transactional sex at a university in Zimbabwe Cult Health Sex 2010 12 8 857 70 10.1080/13691050903471441 20069476

